# Exploring rural Scottish GPs’ migration decisions: a secondary qualitative analysis considering burnout

**DOI:** 10.3399/BJGP.2024.0494

**Published:** 2025-02-11

**Authors:** Helen Ann Latham, Andrew S Maclaren, Johannes H De Kock, Louise Locock, Peter Murchie, Zoë Skea

**Affiliations:** NHS Highland, Inverness, and clinical research fellow, University of Aberdeen, Academic Primary Care Group, Centre for Rural Health, Inverness, UK.; School of Medicine, Medical Sciences and Nutrition, University of Aberdeen, Aberdeen, UK.; NHS Highland, Raigmore Hospital, Inverness, UK and extraordinary professor, School of Psychosocial Research, North-West University, Potchefstroom, South Africa.; Aberdeen Centre for Evaluation, University of Aberdeen, Aberdeen, UK.; Institute of Applied Health Sciences, Academic Primary Care Group, University of Aberdeen, Aberdeen, UK.; Health Services Research Unit, School of Medicine, Medical Sciences and Nutrition, University of Aberdeen, Aberdeen, UK.

**Keywords:** burnout, professional, general practice, Scotland, recruitment, retention, rural health services

## Abstract

**Background:**

The challenges of recruiting and retaining rural GPs are well described. UK data suggest high levels of burnout, characterised by detachment, exhaustion, and cynicism, plays a role in GP turnover. The contrast is engagement with work. There is limited evidence examining the relationship between work engagement and recruitment and retention in rural areas.

**Aim:**

To qualitatively investigate GPs decisions to move or stay in rural areas through exploring areas that can promote work engagement.

**Design and setting:**

This was a secondary analysis of qualitative data with Scottish GPs.

**Method:**

A secondary analysis of 44 semi-structured interviews with GPs from across Scotland was undertaken. Data were analysed thematically and the Areas of Worklife Scale was used to structure data.

**Results:**

Factors associated with burnout were identified and experienced as barriers to moving or staying rurally. Fear of dealing with pre-hospital emergency cases, clinical isolation, and rural training were concerns. Personal factors such as lack of partner employment played a key role in migration decisions. Factors associated with engagement were identified and experienced as facilitators for moving or staying rurally. Professional networks reduced professional isolation and rural GPs valued increased autonomy and time. Many felt being a rural GP was more aligned with their professional values and highly valued the rural lifestyle for themselves and their families.

**Conclusion:**

Our data suggest that factors associated with engagement and burnout can contribute to rural GPs’ migration decisions. We highlight four areas that could promote desirable work environments by mitigating burnout and promoting engagement at work.

## Introduction

**Figure 1.**
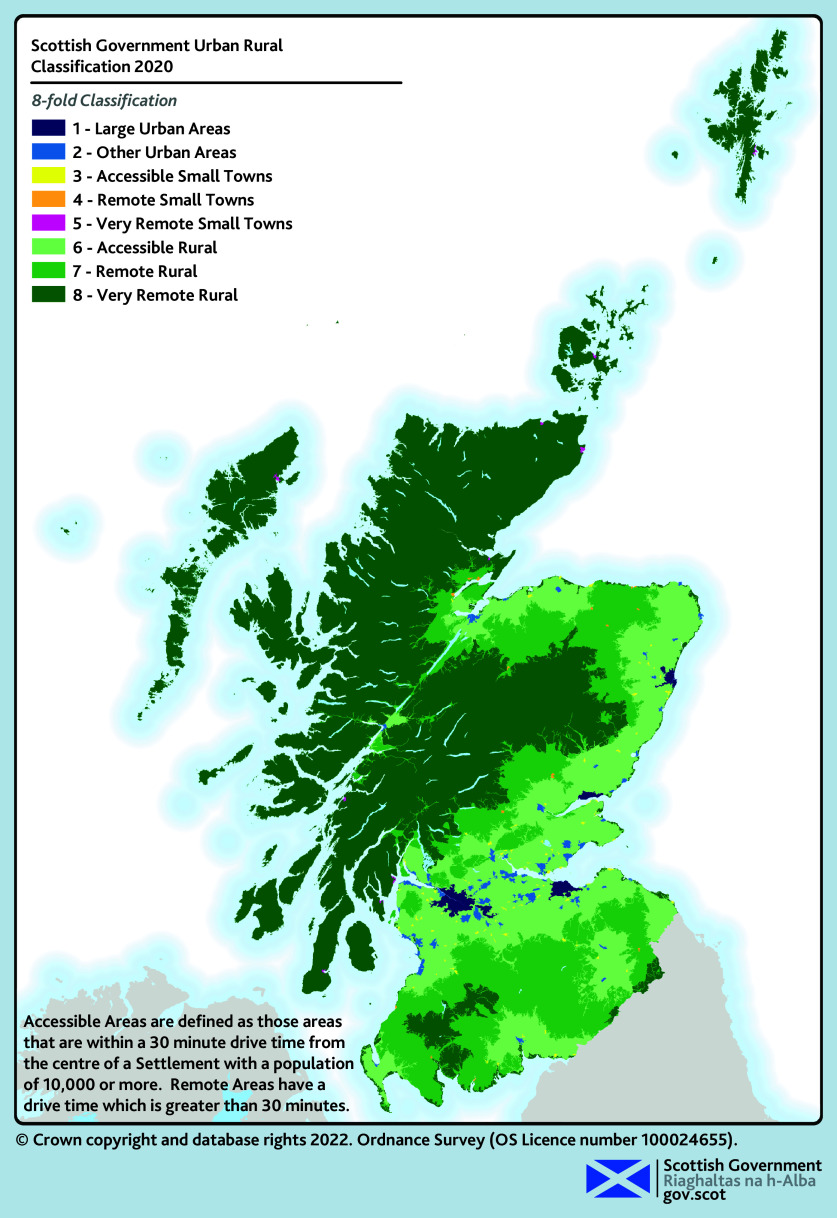
Scottish Government Urban Rural Classification 2020.^29^ © Crown Copyright and database rights, 2022.

Scotland’s rural areas account for 98% of Scotland’s landmass and are home to 17% of the population (see Figure 1).^1^ Access to health care in rural Scotland has been a concern since before the founding of the NHS, with the Dewar Report in 1912 outlining significant health access needs in the Highlands and Islands of Scotland.^2^^,^^3^ The core values of the NHS, to deliver universal and comprehensive health care to all, remain unchanged but delivering this in rural Scotland remains a challenge.^3^

Health care in Scotland is predominantly provided by the NHS, with primary care the first point of contact into the healthcare system and GPs acting as gatekeepers to secondary care.^4^^,^^5^ As such, primary care is central to the functioning of the NHS. General practices in Scotland can be run by the regional NHS health board but are usually independent businesses contracted by the NHS to provide local healthcare services. GPs can work as GP partners, owning and managing the business; or salaried GPs, employed by the health board or GP practice. Others are self-employed, working as GP ‘locums’. GP specialty training takes place in primary and secondary care settings in the NHS and is 3–4 years long.^4^

According to Scottish figures, the highest GP vacancy rates in Scotland occur in the most remote and rural NHS health boards.^6^^–^9 This is against a backdrop of a UK primary care system facing challenges. Overall numbers of GPs have been steadily decreasing and recent research suggest that 39% of GPs in the UK are likely to leave the profession in the next 5 years.^6^^,^^10^ General Medical Council data (2020), reports that of those that left practice, 43% of GPs did so because of burnout.^11^ A recent systematic review also evidences moderate-to-high levels of burnout in the GP workforce.^12^ This is concerning, as high levels of burnout are associated with lower quality of care for patients, lower work satisfaction, productivity loss, and poor staff retention.^13^^–^17

Burnout is defined as ‘a syndrome resulting from failure to control or manage chronic workplace stress’.^18^ It is characterised by three components: exhaustion, cynicism, and detachment.^12^^,^^19^^,^^20^ The contrast to burnout is defined as engagement with work, characterised by employees that are energetic, effective, and involved in their workplace.^21^ Solutions to support healthcare workers’ mental health often focus on strategies to improve individuals’ resilience and ability to cope with stressors.^22^^,^^23^ Although these can be useful, addressing burnout and increasing engagement requires a systemic, organisation-level approach.^22^ There has been considerable research into the factors that protect against burnout and these have been distilled to six core areas of worklife: workload, control, reward, community, fairness, and values.^21^^,^^22^

**Table:** How this fits in

We know that recruiting and retaining GPs in rural Scotland is challenging and that burnout plays a role in GP turnover in the UK, however, we do not know whether promoting work engagement (the contrast to burnout) can influence migration decisions for rural GPs. Our paper provides evidence that aspects of work that increase the risk of burnout and factors that promote the contrast, work engagement, were experienced as influencing migration decisions in rural GPs. We found that core professional values such as continuity of care are important, that professional autonomy is highly valued, and that perceived unfairness in policy and management decisions influence rural GPs. These findings emphasise that creating desirable work environments through promoting engagement with work and the mitigation of burnout can play an important role in recruitment and retention.

The challenges of recruiting and retaining rural healthcare workers have been well documented. Although the focus tends to be on core aspects of the job, recent Scottish Government Chief Scientist Office (CSO)-funded qualitative research into rural recruitment and retention, highlights secondary factors, over and above direct workplace issues, influencing decisions to move to and stay in rural areas.^24^^,^^25^ The research emphasises that competing values are often at play, and that effectively recruiting and retaining healthcare staff in rural communities requires a holistic approach.^24^^,^^25^ This fits with an understanding of burnout as a multidimensional concept, requiring a holistic approach to promote engagement with work and avoid burnout.^21^^,^^22^ A recent framework for rural health workforce stability states that a cohesive team and desirable work environment should underpin recruitment and retention activities.^26^

This paper investigates factors that influence rural GPs’ decisions to move or stay in rural areas by exploring factors that contribute to burnout, and oppositely, factors that can address burnout by promoting engagement with the workplace.

## Method

A secondary analysis of qualitative data^24^^,^^25^^,^^27^ was carried out to explore factors influencing GPs’ engagement with work and decisions to move or stay in rural areas. The original research was funded by the Scottish CSO in 2020, a mixed-methods project investigating recruitment and retention of doctors in rural Scotland that included 56 qualitative interviews.^24^^,^^25^^,^^27^ Using Heaton’s five categories of secondary analysis, this paper is primarily a ‘supplementary analysis’, involving the ‘in-depth investigation of an aspect of the data, that was only partly covered in the original research’.^28^ Secondarily, there are some elements of ‘supra-analysis’, in which ‘new theoretical, empirical or methodological questions are also explored that are distinct from the aims of the original research’.^28^

This paper analysed 44 semi-structured interviews; these are with a subset of GPs in Scotland from a larger dataset of the original 56 interviews.^25^ Participants were GPs or GP registrars working in Scotland, all of whom had experience working rurally. Table 1 gives detail regarding characteristics and geography. GPs were recruited across Scotland, including those currently working in rural places and others who had since moved. Recruitment was done through multiple channels, including email lists of societies (for example, Scottish Rural Medicine Collaborative), GP clusters, and snowball sampling from initial service provision interviews.^25^

**Table 1.** Participant characteristics (*N* = 44)

**Characteristic**	**Participants, *n*** *^a^*
**Gender**	
Female	26
Male	18

**Age, years (estimates)**	
<45	26
>45	18

**Role**	
GP partner	20
GP partner — single handed	2 (1 prior)
Salaried GP	11
Locum GP	7
GP registrar	2
Retired	1
Other: GP in extended role	1

**Location**	
Highland	18
Island	14
Other (Fife/Argyll/Borders/Dumfries & Galloway)	12

**Rural experience**	
Current	42
Prior	2

a n *unless otherwise stated.*

Interviews were conducted by the second author in 2020 via video call or telephone. Each interview was around 1 hour long and nine were ‘service mapping’ interviews with key national informants, as part of an exercise to understand recruitment and retention issues in the Scottish context but included biography of the participants and so included here.^24^^,^^27^ These participants were chosen as they had experience working across a range of locations and were involved in leadership roles. The geographical variability is necessary to increase transferability of the findings.^25^ The remainder were GPs who were either full time, part time, or locum and some recently retired. These interviews focused on participants’ biography, experiences, and perceptions. 25 These interviews were semi-structured with some pre-determined questions regarding their biographical narrative, working life, and career motivations. Conversations were guided by topics brought up by the participant with general themes developed from the initial service mapping interviews that included community, housing, career, and family.^25^

Ethical approval was obtained for the original interviews and participants were explicitly consented for their data to be used for secondary analysis.

### Analysis

A thematic analysis of the original data was undertaken, initially via an inductive approach, using NVivo software to organise codes. Initial themes identified (community support, clinical isolation, professional values, autonomy) were found to map well onto original constructs of the Areas of Worklife Scale (AWS) and this was used to structure data using a more deductive approach. The AWS is a validated method for structuring organisational self-reflection and change, and identifying predictors of burnout.^21^ The six core factors are: workload; control; reward; community; fairness; and values.

Team meetings with the original researchers were undertaken throughout the analysis process with codes added and updated throughout.

## Results

The findings from the data have been organised under the six themes outlined in the AWS. A breakdown of these themes can be found in Box 1.

**Box 1.** Predictors of burnout based on the Areas of Worklife Scale 21^,^^22^

**Workload**	The most discussed aspect of burnout — when job demand exceeds human limits This has a consistent relationship with burnout, mediated by exhaustion
**Control**	Employee’s perceived capacity to influence decisions that affect their work, to exercise professional autonomy, and to gain access to resources necessary to do an effective job Lack of control has been a consistent predictor of burnout
**Reward**	The extent to which reward (financial, social, and intrinsic) is consistent with expectations (including recognition and pride in doing something well) Insufficient recognition and reward can devalue work and worker and result in feelings of inefficacy
**Community**	Overall quality of social interaction A consistent finding through research is that a lively, attentive, responsive community is protective against burnout Lack of support and unresolved conflict increases risk of burnout
**Fairness**	The extent to which decisions at work are perceived as being fair and people are treated with respect
**Values**	This is at the heart of people’s relationship with work and encompasses the ideals and motivations that originally attracted them to the job Mediates the relationship of all the other areas of the AWS (other than workload) Feeling that the work you do is not in keeping with the work that you want to do can increase burnout

*AWS = Areas of Worklife Scale.*

### Workload

Workload in the rural setting was portrayed as different to that in the urban setting. GPs who had worked in urban and rural settings reported being able to spend more time with patients than they could in an urban practice, enabling them to deliver care the way they wanted:

*‘I can be a GP in the style that I enjoy. I can get to the end of the day and remember the decisions I’ve made here because it’s not been so fast moving.’*

(GP with rural and urban role describing rural job, female [F])

There were aspects that were described as increasing workload and pressure. The responsibility of providing clinical care without easy access to secondary care facilities, was experienced by some as an enjoyable challenge when they felt equipped to do so. In contrast, the pressure of ‘being it’ and dealing with emergency situations without adequate support or training to maintain skills, led others to leave the rural setting or avoid it completely:

*‘It’s viewed as a scary place to work because there is an emergency care provision requirement, because our ambulance response times can be long. Long in a city is 20 minutes; we could actually be with a critically sick person for a couple of hours. You really have to be comfortable with dealing with sick people to be in sole charge for that kind of length of time. There are a certain subgroup of GPs who find that fulfilling, and the vast majority of them who run screaming from the concept.’*

(Rural GP, F)

One GP described challenges in training GPs to work in the rural setting:

*‘The other thing I think also it’s the fact that increasingly the training is becoming so urban centric … and there’s just a dichotomy between the two. They were training doctors who can’t work in rural areas because they just haven’t got the skills and they lack the … it’s just too foreign for them and I think that’s another problem as well.’*

(Rural GP partner, F)

Out-of-hours commitments in rural practice were described as creating pressure on doctors and on their families, particularly if there were a lack of colleagues to cover time off. In some cases, the increased workload and pressure led to exhaustion and leaving the rural role:

*‘You know, it was having a huge impact on family life but also personally, I mean, just the exhaustion, you know? We moved there for the lifestyle and, okay, yes, my kids were growing up in a beautiful place, in a good community and all the rest, but if their mother isn’t available to them because she’s exhausted all the time, I mean, that’s not what we wanted either.’*

(Rural GP partner describing previous remote salaried post, F)

### Control

GPs reported valuing autonomy and flexibility and described how this attracted them to general practice as a career, with the perception of having more control and choice over where to live and work than other specialties:

*‘I applied for GP training and had really decided that that was the right path for me at that time, largely because I think it’s the most flexible career.’*

(GP locum, rural and urban, F)

Rural practice was considered by many to offer a greater sense of autonomy than urban practice. They valued the choice to live in a rural setting. They described how small team sizes and time to interact with staff and patients resulted in a greater sense of control in how they delivered care to patients, as described by a rural GP:

*‘So for me it is a bit of a balancing act, you have to prioritise a lot of things, but it, I feel it gives a bit more autonomy to be able to deal with the issues on a day, to shape my day, to be in control about what’s happening in the practice, and I think that, for me that’s a major plus.’*

(Rural GP partner, male [M])

### Reward

The main reward raised by rural GPs was the rural lifestyle that participants valued for themselves and their families. This was an incentive for choosing rural practice, particularly for those with a rural background. Some described this as being more important than the job:

*‘I see the city folk as being deprived of nature and beauty, and lovely walks, and pure fresh air, and sea lochs that you can go swimming in, and actually that to me is more valuable, and that’s why I’m here, I suppose.’*

(Salaried GP, F)

*‘I think with two little ones, the idea of them having the opportunity to grow up in an environment with lots more outdoor space, outdoor activities, that sort of thing, that’s more what was appealing rather than having a keen interest in sort of pre-hospital medicine or something specific in relation to remote and rural practice.’*

(Rural GP registrar, M)

Most GPs did not consider financial reward as an incentive to move or stay rurally. Doctors described valuing quality of life over higher income. Nevertheless, when explored further, finances did influence rural GPs’ wellbeing and job choices. GPs described feeling undervalued when they were paid less or the same as those with less responsibility, or when funding allocations to rural practice were deemed to undervalue the role of the rural GP:

*‘I think it’s really important that everybody feels valued financially and I think it’s very hard if you’re working and you come across somebody who’s doing the same, more or less, than you, and they’re earning more than you.’*

(GP with rural and urban role, F)

### Community

Community, or the relationships that surround a role, played a major role in participants’ work choices and were often more important than the job itself. Broadly these findings have been split into professional community (the relationships at work) and personal community (family, friends, rural community).

#### Professional.

Participants experienced rural working environments as being more personal and supportive than urban equivalents. However, clinical isolation was a concern for many, owing to lack of colleagues or lack of nearby secondary care facilities. This led some rural GPs to feel unsupported and overworked and for others the fear of clinical isolation was a disincentive to working rurally:

*‘Ultimately for me, I left because I was finding single handed practice too isolating, in a nutshell.’*

(Rural GP partner describing previous remote job, F)

GPs involved in professional networks described how this enabled them to feel connected and supported by a group of peers. This played a part in counteracting clinical isolation. These networks could be regular meetings between rural GPs, rural GPs and secondary care, or formal connections with larger practices:

*‘There was a lot of meetings for rural GPs, and I attended quite a lot of those when I was in (rural practice), and I think things like that really kind of, I guess make you feel a bit more part of the community and feel a bit more supported in some of the kind of decisions that you’re making in those places, so I think things like that were really like positive, and I think would give me a bit of confidence, if I was wanting to go to those more rural practices that you kind of, you’re not so isolated.’*

(Salaried GP, rural/urban, F)

#### Personal.

Being part of a supportive rural community was described as a positive for doctors. However, a lack of separation between personal and work life in a small community led some to avoid the rural setting. Participants described a lack of anonymity and a fear that any clinical mistakes would have greater consequences:

*‘How do you settle into a community, particularly if you’re only meeting people in that role, it’s really difficult. People then tend to see you as “the doctor”. That’s still a challenge, it’s not as prevalent here but even there, in a small community, you know, we used to call it the Spar ward round because people would come up to you in the aisles when you’re there … and start talking about something completely inappropriate!’*

(Rural GP partner, F)

Support and connection with family and friends was stated as a reason to move or stay in a job — whether it was urban or rural. Rural infrastructure had an impact on GP’s working choices, whether it was access to housing, travel, schooling, or lack of spouse employment. This influenced doctors differently depending on their circumstances. Some moved to provide their family with the rural lifestyle they valued, others left rural life as they were unable to care for their family — whether it was being close to older relatives, choice of schooling for children, or employment for their partner. One GP described the impact of this on their ability to recruit:

*‘I’ve seen that several times, and I’ve seen couples where the guy’s been interested because it’s such a unique job, but the spouse has turned it down, first of all because you couldn’t give them a job, cause these days you have to find two jobs, but also they looked at the schools and school rolls are all falling, they’re going down, they’re amalgamating the schools.’*

(Retired GP partner, remote/island, F)

### Fairness

Many rural doctors, particularly those in partnership roles, described an environment of feeling unsupported by policymaking and management structures. Policies that were felt to undervalue or disregard the rural GP role, were considered unfair and led to frustration. This could exacerbate a sense of being remote. In contrast, management that was perceived as supportive of rural general practice was considered a great advantage:

*‘We’re very remote from the people that make decisions and increasingly if you write to them they, they ignore you, they don’t even write back and say, “Sod off”, they just ignore you.’*

(GP partner, remote/island, M)

Pensions were a factor for GPs who were nearing retirement, with tax policies perceived as being unfair and penalising them for working. This led some to reduce their working hours or retire earlier than planned.

### Values

There were aspects of rural general practice that resonated with GPs’ professional identity and core values. Rural practice was perceived to be a space where participants could practise general practice the way they wanted to, as articulated by this GP:

*‘From a professional point of view, it would be an ability to influence a small area in terms of realistic, humanistic, relationship-centred health care with a view to community wellbeing, which feels unattainable in a town or city practice.’*

(GP partner, remote/island, M)

A core aspect of the GP identity involved continuity of care and the ability to positively influence a community. This was a factor attracting doctors to move and stay in rural areas. Continuity of care increased job satisfaction and was considered more efficient with improved clinical care:

*‘I mean, job satisfaction, I think, is right up there. You got continuity with the patients, that’s probably the single biggest factor, you’re not rushing around seeing a different patient every day, you’re seeing the same patients and you get to know them, which makes things far more efficient.’*

(Rural GP partner describing the benefits of rural practice, M)

## Discussion

### Summary

Our study outlines features of rural general practice in Scotland that align with the constructs of the AWS that are associated with burnout, but also with engagement with work — the ‘antidote’ to burnout.

Factors aligned with the AWS associated with burnout were identified as workload, reward, community, and fairness (see Box 1) and were experienced as barriers to moving or staying rurally. Out-of-hours commitments, clinical isolation, lack of anonymity outside work and fear of dealing with pre-hospital emergency cases without adequate experience or support were raised as factors that negatively had an impact on rural GPs and were experienced as barriers to moving and staying rurally. Participants described challenges in training rural GPs to develop and maintain necessary skills. Although financial incentives were not considered major drivers, finances did influence rural GPs, when experienced as unfair or undervaluing their rural role. Lack of colleagues and vacant roles were reported to increase clinical isolation and exhaustion. Personal factors, such as lack of partner employment or inadequate infrastructure, played a key role in decisions to move or stay and for many were more important than the job itself. Many rural doctors described an environment of feeling unsupported and undervalued by policymakers.

Factors aligned with the AWS associated with the contrast to burnout — engagement with work — were identified as community, autonomy, workload, and values. These were also experienced as facilitators to moving to or staying rurally. GPs that felt equipped to deliver pre-hospital urgent care enjoyed this aspect of the rural role and professional networks reduced professional isolation for those that had access to them. Participants valued the increased autonomy they experienced rurally, and many enjoyed more time with patients than in an urban practice and a more supportive working environment. Many described rural general practice as being more aligned with their professional values of providing continuity of care for patients and taking ownership for positive change in their community, and their personal values of enabling a rural lifestyle for themselves and their families.

### Strengths and limitations

This paper adds to the literature exploring recruitment and retention factors for rural GPs and provides a perspective using evidence from organisational psychology regarding burnout and engagement. This is in keeping with calls for organisational change within the health system and adds to a dearth of data exploring the relationship between burnout, engagement, and retention in rural general practice.^11^^,^^22^^,^^30^^–^33 Although the purpose of qualitative research is not to seek statistical representativeness, maximum variation sampling aims to provide insights into a diverse range of experiences. This study draws from a wealth of qualitative data from GPs spread across Scotland in a range of different roles, providing both breadth and depth of understanding. Being a secondary analysis and with data focused on the experiences of GPs in Scotland regarding rural Scottish GP workplaces findings may not be generalisable to all settings. However, our data echoed findings from literature in other countries and the factors raised in our paper can be relevant in other rural settings.^26^^,^^34^^,^^35^

### Comparison with existing literature

Although there have been recommendations to improve recruitment and retention of rural GPs and strategies to reduce burnout in healthcare workers, systematic reviews of both topics indicate a lack of high-quality evaluation of interventions.^22^^,^^34^^,^^36^^–^41 The strongest evidence for recruitment is that rural background and rural exposure in training is associated with improved retention and recruitment of rural GPs.^39^^,^^41^^–^44 Our study highlights four key areas, in keeping with previous literature, that can mitigate burnout and promote engagement at work and could influence GP’s decisions to move or stay in rural areas. These are:^32^^,^^33^

- define the core role of rural primary care;
- community participation;
- enable rural training pathways; and
- fairness.

The first key area, define the core role of rural primary care, is aligned with Abelsen *et al*’s cross-country framework for recruiting and retaining rural healthcare workers and the need to recognise the unique issues involved in rural and remote settings and assessing the community needs.^21^^,^^26^ As the role of the GP adapts to system pressures, defining and protecting the core values and role of rural primary care has potential to increase professional autonomy and identity as well as identifying the training needs of rural practitioners.^19^^,^^21^ Our paper suggests that rural work is experienced as providing GPs with greater professional autonomy and the chance to connect with their professional values, particularly in providing continuity of care. Continuity of care is recognised to improve clinical outcomes and is valued by patients; this study suggests that it is also highly valued by GPs.^45^^–^48 Policies that focus solely on outcomes such as workload and access to care risk eroding these professional values.^49^ The Deep End Project for GPs working in deprived practices in Scotland is a good example of defining a role and creating a professional network that promotes autonomy, identity, and purpose and enables GPs to advocate for their patients.^50^ This could also strengthen and develop professional networks that have potential to mitigate isolation.

The second key area relates to community participation. Building on previous research, this paper suggests the drivers for retaining and recruiting rural GPs may be located not only within the health system, but also within its community context.^24^^,^^25^^,^^27^^,^^34^ Family and personal needs of doctors play a major role and can be stronger drivers than the job itself.^34^^,^^35^ With a Scottish GP workforce that is predominantly female (62%), the impact of career on families is likely to play an increasing role.^6^^,^^31^^,^^51^ Our paper also suggests the major reward associated with a rural job is the rural lifestyle, indicating that many rural GPs are likely to be moving for place rather than job. Involving rural communities in the process of recruitment, community integration, and support for spouses and families has potential to increase engagement with work and in turn improve retention.^26^

The third key area relates to enabling rural training pathways. Obtaining and maintaining rural skills was described as a challenge and fear of delivering urgent care and clinical isolation are a concern. Accessible pathways to develop and maintain rural skills could ensure that GPs feel safe and equipped to deliver care in rural settings.^9^^,^^26^ Rural GP training and rural fellowships for newly qualified GPs are available in Scotland but our data suggests that downstream options for GPs later in their career remain a challenge.^52^ Evaluation of existing interventions are recommended to build an evidence base for policymakers addressing recruitment and retention in rural general practice.

The final key area relates to fairness. It is of concern that rural GPs felt generally unsupported by senior management and government, in keeping with evidence from UK studies that NHS staff do not feel their work is valued by government.^53^ Our data also suggests that although financial incentives may not be the major driver in migration choices, rural GPs feel undervalued when salary and financial allocations are considered unfair. This reflects evidence from a recent systematic review of NHS staff, that poor pay influences job satisfaction, but also that increase in pay alone does not improve retention.^54^ Rural primary care has unique needs and pressures and recognition of this in policy decisions can enable a fair future for rural primary care.

### Implications for research and practice

This paper indicates positive aspects of rural practice in Scotland that could promote engagement with work but also highlights aspects that could increase the risk of burnout. Our data suggests these also play a role in GPs’ decisions to move or stay in rural areas. Further studies could take note of the four areas highlighted by our data that could mitigate burnout and promote engagement at work. These are: defining the core values and role of rural primary care; community participation; enabling rural training pathways; and fairness — considering rural primary care needs in policy decisions. Scotland has a heritage of innovative interventions addressing GP workforce issues including rural track GP fellowships and the Deep End Project in urban deprived GP practices and there is great potential for the future of rural general practice in Scotland.^50^^,^^55^
